# The complete mitogenome sequence of the hawk moth, *Theretra latreillii* subsp. *lucasii* (Lepidoptera: Sphingidae) from Zhejiang Province, China

**DOI:** 10.1080/23802359.2021.1934152

**Published:** 2021-06-07

**Authors:** Qiaoying Lu, Hongwei Yao, Jinming Zhang, Hongxing Xu, Caiying Jiang

**Affiliations:** a College of Life Sciences and Medicine, Zhejiang Sci-Tech University, Hangzhou, Zhejiang, China; b Zhejiang Provincial Key Lab of Biology of Crop Pathogens and Insects, Ministry of Agriculture Key Lab of Molecular Biology of Crop Pathogens and Insects, Institute of Insect Sciences, Zhejiang University, Hangzhou, Zhejiang, China; c State Key Laboratory for Managing Biotic and Chemical Threats to the Quality and Safety of Agro-products, Institute of Plant Protection and Microbiology, Zhejiang Academy of Agricultural Sciences, Hangzhou, Zhejiang, China

**Keywords:** mitogenome, Sphingidae, *Theretra latreillii lucasii*

## Abstract

The sphingid, *Theretra latreillii* subsp. *lucasii* is a common hawk moth distributed in southeast Asia and Australian regions. Although barcode analyses have been published, its complete mitogenome sequence has not been deciphered. In this study, the complete mitogenome of *T. latreillii lucasii* (GeneBank accession no. MW539688) was sequenced using Illumina HiSeq X Ten system for mitogenome-based phylogenetic analysis. The mitogenome was 15,354 bp in length and comprises 13 protein-coding genes (PCGs), two ribosomal RNA (rRNA) genes, and 22 transfer RNAs (tRNAs) with the typical gene order and orientation of Sphingidae mitogenomes. The nucleotide composition of majority strand is 41.2% for A, 7.4% for G, 12.0% for C, and 39.4% for T, with an A + T content of 80.6%. Phylogenetic analysis using the 13 PCGs fully resolved *T. latreillii lucasii* in a clade with *T. japonica*, *Macroglossum stellatarum*, and *Ampelophaga rubiginosa*, with high nodal support both by Bayesian inference and maximum-likelihood methods, forming the Macroglossini monophyletic group.

The hawk moths (Lepidoptera: Sphingidae) have attracted the attention of researchers for a long time with their slender body shape and agile flying behavior as adults, as well as their large size and realistic mimicry as larvae (Abrera 1986; Kawahara et al. 2009; Rafi et al. 2014). There are more than 1400 recognized species in some 200 genera of Sphingidae, which are found on every continent except Antarctica (Kawahara et al. 2009; van Nieukerken et al. 2011). Some species play a vital role as pollinators in many wild ecosystems (Alexandersson and Johnson 2002). Others are important agricultural or ornamental insect pests (Sambath 2011; Rafi et al. 2014; Rougerie et al. 2014). Studies of the genetic diversity of Sphingidae have contributed to the understanding of their adaptive evolutionary mechanisms (Hundsdoerfer et al. 2005; Barth et al. 2018). To date, complete mitogenome sequences of Sphingidae are available from several species, *Manduca sexta* (EU286785) (Cameron and Whiting 2008), *Sphinx morio* (KC470083) (Kim et al. 2013), *Notonagemia analis scribae* (KU934302) (Kim et al. 2016), *Ampelophaga rubiginosa* (KT153024) (Xin et al. 2017), *Theretra japonica* (MG655620) (Li, Lin, et al. 2018), *Macroglossum stellatarum* (MG747645) (Li, Zhang, et al. 2018), *Psilogramma increta* (MF974243) (Li, Zhang, et al. 2018), and *Parum colligate* (MG888667) (Li et al. 2019). In this study, we sequenced the complete mitogenome of the sphingid *Theretra latreillii* subsp. *lucasii* (Walker), distributed in the Oriental and Australian regions (Rafi et al. 2014) to determine its mitogenome structure and phylogenetic relationship to other Sphingidae.

Larvae of *T. latreillii lucasii* were collected on *Tetrastigma hemsleyanum* Diels et Gilg at Ruoheng (28°23′33″ N, 121°29′45″ E), Zhejiang Province, China, in June 2020, and were reared on fresh leaves of *T. hemsleyanum* until eclosion in the insectary at 25 ± 1 °C, RH 60 ± 5% and L:D = 14:10 h. Adult specimen was deposited at Institute of Insect Sciences, Zhejiang University (http://www.cab.zju.edu.cn/iae/, Hongwei Yao, hwyao@zju.edu.cn) under the voucher number ZJUIS2020-011. Total genomic DNA was extracted from the larvae of *T. latreillii lucasii* using phenol–chloroform method, and was sequenced using Illumina HiSeq X Ten system with the strategy of 150 paired-ends reading. The mitogenome was annotated using the Geneious 11.0.4 version and MITOS Web Server (Bernt et al. 2013).

The mitogenome of *T. latreillii lucasii* is 15,354 bp in length (GenBank accession no. MW539688) and contains 13 protein-coding genes (PCGs), 22 transfer RNA (tRNA) genes, two ribosomal RNA (rRNA) genes, and one non-coding region (the A + T-rich region). The genome size of *T. latreillii lucasii* is consistent with the 15,312 bp reported for *T. oldenlandiae* (Wang et al. 2020) and 15,399 bp for *T. japonica* (Li, Lin, et al. 2018). There are 23 genes encoded on the majority stand, and the remaining 14 genes on the minority strand. The gene order and orientation are consistent to *T. japonica* and other Sphingidae. The nucleotide composition in majority strand of *T. latreillii lucasii* mitogenome is 41.2% for A, 7.4% for G, 12.0% for C, and 39.4% for T, with an A + T content of 80.6%. All 13 PCGs initiate with ATN start codons and terminate mostly with the TAA stop codon. The 22 tRNA genes varied from 64 to 69 bp in length. The gene arrangement of the tRNA cluster, trnM-trnI-trnQ between the A + T-rich region and ND2, is identical to that of Sphingidae and other ditrysian Lepidoptera (Cameron and Whiting 2008; Kim et al. 2013, 2016). The large ribosomal gene (rrnL) was located between trnL1 and trnV with a length of 1381 bp, whereas the small ribosomal gene (rrnS) was located between trnV and the A + T-rich region with a length of 761 bp.

Phylogenetic analysis of *T. latreillii lucasii* with 14 other species in three families of Bombycoidea was performed by using nucleotide sequences of 13 PCGs from their mitogenomes. Two species of Noctuoidea, *Spodoptera litura* and *Helicoverpa armigera*, were designated as outgroups. The sequences were aligned by using MAFFT v7.271, and the phylogenetic tree was constructed by CIPRES (https://www.phylo.org/) using MrBayes on XSEDE and IQTree on XSEDE with the best-fit nucleotide substitution model GTR + F+I + G4 determined by ModelFinder (Kalyaanamoorthy et al. 2017) with bootstrap 1000 (Figure 1). The analysis showed that *T. latreillii lucasii* and *T. japonica* were clustered in the *Theretra* clade. The three families of Bombycoidea were grouped in different clades, as well as the three tribes of Sphingidae. The result of phylogenetic analysis was consistent with other results from the analyses based on morphological characters and mitochondrial sequences before (Rougerie et al. 2014; Kim et al. 2016; Wang et al. 2020). This study enriches the Sphingidae genetic database, and also further clarifies our understanding of the phylogenetic relationships of the Sphingidae.

**Figure 1.**
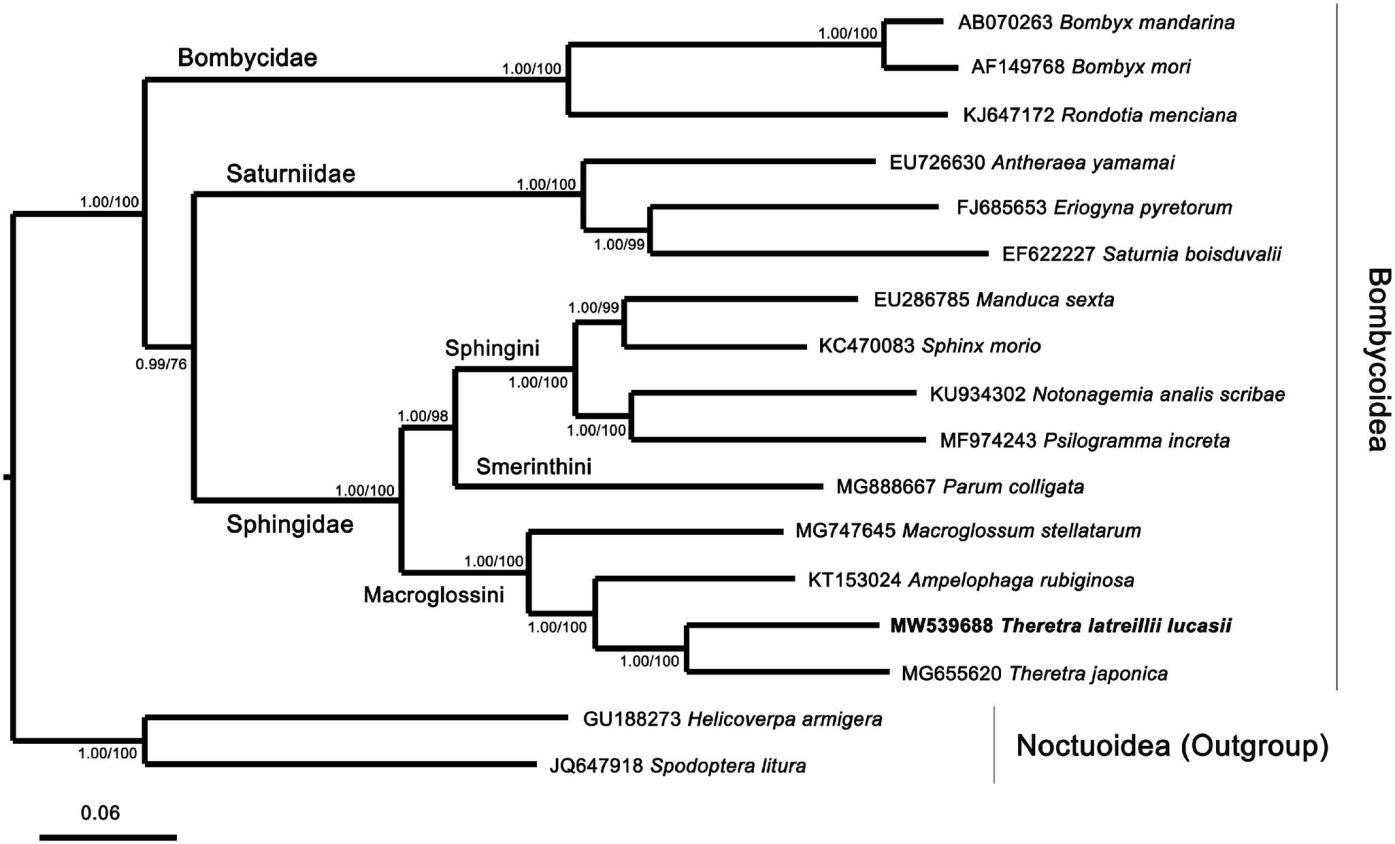
Phylogenetic tree of Bayesian inference (BI) and maximum-likelihood (ML) methods using matrixes of 13 PCGs in mitogenomes of 15 representative species classified in the Bombycoidea and two species of Noctuoidea serving as outgroups. The numbers at each node are Bayesian posterior probabilities by BI analysis (first value) and bootstrap percentages of 1000 pseudoreplicates by ML analysis (second value). The scale bar indicates the number of substitutions per site.

## Data Availability

The genome sequence data that support the findings of this study are openly available in GenBank of NCBI (https://www.ncbi.nlm.nih.gov/) under the accession no. MW539688. The associated BioProject, SRA, and BioSample numbers are PRJNA721802, SRP314974, and SAMN18739530, respectively.

## References

[CIT0001] Abrera DB. 1986. Sphingidae mundi: hawk moths of the world. Faringdon (UK): E. W. Classey Ltd.; p. 226.

[CIT0002] Alexandersson R, Johnson SD. 2002. Pollinator-mediated selection on flower-tube length in a hawkmoth-pollinated *Gladiolus* (Iridaceae). Proc R Soc Lond B. 269:631–636. 10.1098/rspb.2001.1928 PMC1690929 11916480

[CIT0003] Barth MB, Buchwalder K, Kawahara AY, Zhou X, Liu S, Krezdorn N, Rotter B, Horres R, Hundsdoerfer AK. 2018. Functional characterization of the *Hyles euphorbiae* hawkmoth transcriptome reveals strong expression of phorbol ester detoxification and seasonal cold hardiness genes. Front Zool. 15:20. 29743927 10.1186/s12983-018-0252-2 PMC5930835

[CIT0004] Bernt M, Donath A, Jühling F, Externbrink F, Florentz C, Fritzsch G, Pütz J, Middendorf M, Stadler PF. 2013. MITOS: improved *de novo* metazoan mitochondrial genome annotation. Mol Phylogenet Evol. 69(2):313–319. 22982435 10.1016/j.ympev.2012.08.023

[CIT0005] Cameron SL, Whiting MF. 2008. The complete mitochondrial genome of the tobacco hornworm, *Manduca sexta*, (Insecta: Lepidoptera: Sphingidae), and an examination of mitochondrial gene variability within butterflies and moths. Gene. 408(1–2):112–123. 18065166 10.1016/j.gene.2007.10.023

[CIT0006] Hundsdoerfer AK, Kitching IJ, Wink M. 2005. A molecular phylogeny of the hawkmoth genus *Hyles* (Lepidoptera: Sphingidae, Macroglossinae). Mol Phylogenet Evol. 35(2):442–458. 15804414 10.1016/j.ympev.2005.02.004

[CIT0007] Kalyaanamoorthy S, Minh BQ, Wong TKF, von Haeseler A, Jermiin LS. 2017. ModelFinder: fast model selection for accurate phylogenetic estimates. Nat Methods. 14(6):587–589. 28481363 10.1038/nmeth.4285 PMC5453245

[CIT0008] Kawahara AY, Mignault AA, Regier JC, Kitching IJ, Mitter C. 2009. Phylogeny and biogeography of hawkmoths (Lepidoptera: Sphingidae): evidence from five nuclear genes. PLoS One. 4(5):e5719. 19492095 10.1371/journal.pone.0005719 PMC2683934

[CIT0009] Kim MJ, Choi SW, Kim I. 2013. Complete mitochondrial genome of the larch hawk moth, *Sphinx morio* (Lepidoptera: Sphingidae). Mitochondrial DNA. 24(6):622–624. 23452242 10.3109/19401736.2013.772155

[CIT0010] Kim MJ, Kim JS, Kim I. 2016. Complete mitochondrial genome of the hawkmoth *Notonagemia analis scribae* (Lepidoptera: Sphingidae). Mitochondrial DNA Part B. 1(1):416–418. 33473503 10.1080/23802359.2016.1176883 PMC7800934

[CIT0011] Li J, Hu K, Zhao Y, Lin R, Zhang Y, Li Y, Huang Z, Peng S, Geng X, Zhang H, et al. 2019. Complete mitogenome of *Parum colligata* (Lepidoptera: Sphingidae) and its phylogenetic position within the Sphingidae. Zootaxa. 4652(1):126–134. 10.11646/zootaxa.4652.1.6 31716886

[CIT0012] Li J, Lin RR, Zhang YY, Hu KJ, Zhao YQ, Li Y, Huang ZR, Zhang X, Geng XX, Ding JH. 2018. Characterization of the complete mitochondrial DNA of *Theretra japonica* and its phylogenetic position within the Sphingidae (Lepidoptera: Sphingidae). Zookeys. 754:127–139. 10.3897/zookeys.754.23404 PMC5945705 29755260

[CIT0013] Li J, Zhang Y, Hu K, Zhao Y, Lin R, Li Y, Huang Z, Zhang X, Geng X, Ding J. 2018. Mitochondrial genome characteristics of two Sphingidae insects (*Psilogramma increta* and *Macroglossum stellatarum*) and implications for their phylogeny. Int J Biol Macromol. 113:592–600. 29501752 10.1016/j.ijbiomac.2018.02.159

[CIT0014] Rafi MA, Sultan A, Kitching IJ, Pittaway AR, Markhasiov M, Khan MR, Naz F. 2014. The hawkmoth fauna of Pakistan (Lepidoptera: Sphingidae). Zootaxa. 3794:393–418. 24870331 10.11646/zootaxa.3794.3.4

[CIT0015] Rougerie R, Kitching IJ, Haxaire J, Miller SE, Hausmann A, Hebert PD. 2014. Australian Sphingidae – DNA barcodes challenge current species boundaries and distributions. PLoS One. 9(7):e101108. 24987846 10.1371/journal.pone.0101108 PMC4079597

[CIT0016] Sambath S. 2011. Studies on the sphingid fauna (Lepidoptera: Heterocera: Sphingidae) of Dalma Wildlife Sanctuary, Jharkhand. Rec Zool Surv India. 111(1):25–30.

[CIT0017] van Nieukerken EJ, Kaila L, Kitching IJ, Kristensen NP, Lees DC, Minet J, Mitter C, Mutanen M, Regier JC, Simonsen TJ, Wahlberg N, Yen S-H, Zahiri R, Adamski D, Baixeras J, Bartsch D, Bengtsson BÅ, Brown JW, Bucheli SR, Davis DR, de Prins J, de Prins W, Epstein ME, Gentili-Poole P, Gielis C, Hättenschwiler P, Hausmann A, Holloway JD, Kallies A, Karsholt O, Kawahara AY, Koster S, Kozlov M, Lafontaine JD, Lamas G, Landry J-F, Lee S, Nuss M, Park K-T, Penz C, Rota J, Schintlmeister A, Schmidt BC, Sohn J-C, Solis MA, Tarmann GM, Warren AD, Weller S, Yakovlev RV, Zolotuhin VV, Zwick A. 2011. Order Lepidoptera Linnaeus, 1758. Zootaxa, 3148:212–221.

[CIT0018] Wang X, Zhang H, Qu WY, Huang YX. 2020. The complete mitochondrial genome sequence of the hawk moth, *Theretra oldenlandiae* (Lepidoptera: Sphingidae). Mitochondrial DNA Part B. 5(1):978–979. 33366835 10.1080/23802359.2020.1719929 PMC7748810

[CIT0019] Xin ZZ, Yu L, Zhu XY, Wang Y, Zhang HB, Zhang DZ, Zhou CL, Tang BP, Liu QN. 2017. Mitochondrial genomes of two Bombycoidea insects and implications for their phylogeny. Sci Rep. 7(1):6544. 28747720 10.1038/s41598-017-06930-5 PMC5529375

